# Transcriptomic profiling of the central amygdala in a rat model of diabetes-associated neuropathic pain

**DOI:** 10.1038/s41597-026-07292-2

**Published:** 2026-04-20

**Authors:** Xiaomin Nie, Jinling Yan, Xiangjie Song, Jiayin Yue, Aijun Jiang, Sijia Chu, Wei Wang

**Affiliations:** 1 Department of Endocrinology and Metabolism, Centre for Leading Medicine and Advanced Technologies of IHM, The First Affiliated Hospital of USTC, Division of Life Sciences and Medicine, University of Science and Technology of China, Hefei, 230001 China; 2 Core Facility Centre for Life Science, Division of Life Sciences and Medicine, University of Science and Technology of China, Hefei, 230026 China

## Abstract

Painful diabetic neuropathy (PDN) is a debilitating complication of diabetes, yet its central nervous system pathogenesis remains poorly understood. We investigated transcriptomic alterations in the central amygdala (CeA) in a diabetic rat model exhibiting neuropathic pain behavior. Male Sprague-Dawley rats were intraperitoneally injected a single dose of streptozotocin to induce diabetes. Six weeks following administration, the development of neuropathic pain was confirmed by the von Frey filament test. Subsequently, RNA sequencing of the CeA tissues was performed. This dataset provides a transcriptomic resource of the CeA in a rat model of diabetes-associated neuropathic pain, which is publicly available to facilitate research into the central mechanisms of PDN.

## Background & Summary

Diabetes has become a global health crisis^1^, with more than half of patients developing neuropathy and up to one-third progressing to painful diabetic neuropathy (PDN)^2^. PDN typically originates in the distal limbs and manifests as symmetrical burning, electric shock, or acupuncture-like pain that is often more severe at night. This condition is frequently accompanied by allodynia and hyperalgesia. PDN presents with heterogeneous phenotypes. The characteristics of patients who will encounter PDN are currently unpredictable^3^. The clinical treatment of PDN relies primarily on analgesics; however, even with polypharmacy, only approximately one-third of patients achieve effective pain relief^4^. The variability in drug response and the limited efficacy of treatments^3^ underscore the need for further exploration into the underlying mechanisms of PDN.

The pathogenesis of PDN involves both peripheral and central mechanisms^5^. Peripheral factors include glycolipid toxicity, which damages nerves via vascular and metabolic pathways, such as nerve hypoxia, the polyol pathway, and the activation of protein kinase C enzymes. Central mechanisms involve alterations at both the spinal cord and brain levels, where structural and functional changes contribute to the amplification of nociceptive processing. Neuroimaging studies have demonstrated that structural alterations in the thalamus, hypothalamus, primary somatosensory cortex, ventrolateral periaqueductal gray and amygdala may contribute to the sensory phenotypes of PDN^6–9^. Notably, the latter two regions are recognized as key nodes in the descending pain modulatory system (DPMS)^10^. More than half of patients who experience chronic neuropathic pain also suffer from mood disorders, such as depression and anxiety, creating a mutually reinforcing cycle that exacerbates both conditions^11^. These findings highlight the importance of central mechanisms and suggest that the brain nuclei involved in processing pain and emotion should be focal points for further investigation.

The amygdala, a complex cluster of nuclei within the limbic system, has traditionally been associated with emotional and motivational processes. Recent studies have further identified the amygdala as a neural basis for emotional responses to pain^12^. Specifically, the central amygdala (CeA), often referred to as the “nociceptive amygdala”, houses numerous neurons that process pain-related information. The CeA can amplify or inhibit pain signals through opposing changes in the excitability of various subpopulations of GABAergic neurons, functioning as a “rheostat” for pain modulation^13^. Additionally, somatostatin-expressing neurons in the CeA may mediate comorbid depressive symptoms in chronic pain^14^. Long-term depression (LTD) in the lateral/basolateral nucleus of the amygdala (LA/BLA)-CeA synapse pathway underlies the comorbid aversive and depressive symptoms of neuropathic pain^15^. Consequently, the CeA is a critical target for the treatment of chronic neuropathic pain and pain-related mood disorders.

Despite the limited research on the connection between PDN and the amygdala, existing studies have identified a reduction in white matter connectivity in the amygdala of PDN patients^9^. Neuroimaging studies in diabetic rats have revealed significant activation of brain regions involved in pain processing, including the amygdala^16^. Previous studies have indicated a potential association between the amygdala and PDN from a structural perspective. However, a comprehensive transcriptomic profile of the CeA in PDN is still lacking. In this study, we performed RNA sequencing on CeA tissues to generate a publicly accessible transcriptomic dataset from a rat model of diabetes-associated neuropathic pain. This resource aims to facilitate the exploration of molecular mechanisms in PDN.

## Methods

### Animals

Male Sprague-Dawley rats aged 6 weeks and weighing 120–140 g were acquired from GemPharmatech (T005818, Nanjing, China). The rats were group-housed under a 12 h light/dark schedule (lights from 07:00–19:00) with food and water ad libitum.

### Ethics statement

All animal experiments were conducted in strict compliance with international ethical guidelines and relevant regulations. The study was approved by the Institutional Animal Care and Use Committee (IACUC) of the University of Science and Technology of China (Approval No.: USTCACUC26120122066). All procedures were performed at the Animal Facility Center of the University of Science and Technology of China, and strictly followed the Guide for the Care and Use of Laboratory Animals (8th Edition) for housing, husbandry, anesthesia, surgery, and general animal care. Euthanasia was performed in accordance with the AVMA Guidelines for the Euthanasia of Animals (2020 Edition). All efforts were made to minimize the number of animals used and to avoid unnecessary discomfort or harm. The design and reporting of animal experiments also adhered to the ARRIVE Guidelines 2.0 to ensure transparency and reproducibility.

### Experimental protocol

To induce diabetes, a single intraperitoneal injection of 60 mg/kg streptozotocin (STZ) was administered to the rats that had fasted for at least four hours but were allowed access to water. Prior to injection, STZ was quickly dissolved in sodium citrate buffer (pH 4.5, 50 mM) at a final concentration of 20 mg/ml, and the injection was completed within five minutes. An equal volume of sodium citrate buffer (vehicle) was injected into the age-matched control rats. After the injection, the rats were provided free access to food and 10% (w/v) sucrose water for 48 hours, after which the sucrose solution was replaced with regular water. A glucometer (Roche, Switzerland) was used to measure blood glucose levels in tail vein blood samples from 6-hour fasted rats to confirm STZ-induced hyperglycemia. Rats with blood glucose levels exceeding 16.7 mmol/l were selected for this study. At the end of each experiment, blood glucose levels and body weights were recorded. According to the modeling method and blood glucose levels, the rats were ultimately divided into a diabetes group (n = 8) and a control group (n = 8).

### Von Frey filament test

Six weeks post-STZ injection, calibrated von Frey filaments were used to assess the mechanical withdrawal threshold of the rats. To acclimatize the animals to the testing environment, each rat was individually placed in a transparent plastic chamber on a wire mesh grid for at least 30 min. The mechanical withdrawal threshold was then tested via von Frey filaments applied to the mid-plantar surface of bilateral hind paws separately. The pressure from the von Frey filament was gradually increased until a positive response was observed, as indicated by the rat withdrawing or licking its paw. The withdrawal threshold was assessed every 10 minutes, and the mean withdrawal threshold was calculated from three applications. The average value of the bilateral hind paw thresholds was calculated as the final mechanical withdrawal threshold for each rat. The experimenters were blinded to group identity during the testing and quantitative analyses.

### Central amygdala tissue collection

The rats were deeply anesthetized and euthanized by an intraperitoneal injection of a lethal dose of pentobarbital sodium (150 mg/kg). Death was verified by the cessation of respiration, heartbeat, and loss of pedal withdrawal reflexes. After death was verified, brain tissues were dissected and collected for further processing. The brains were placed in cold, oxygenated artificial cerebrospinal fluid (aCSF) (95% O2/5% CO2, 124 mM NaCl, 24 mM NaHCO3, 12.5 mM glucose, 2.5 mM KCl, 1.25 mM NaH2PO4, 2 mM CaCl2, 1 mM MgCl2, 5 mM HEPES, pH 7.4, 300–310 mOsm) and sliced into 300-μm-thick coronal sections via a vibratome (Leica VT1000 S). Bilateral CeA was microdissected under a dissecting microscope in accordance with the rat brain stereotaxic coordinates (coordinates from bregma: anterior/posterior, −2.6 mm; medial/lateral, ± 4.2 mm; dorsal/ventral, −6.1 mm) using 14- or 12-gauge metal needles^17^. Tissue samples were collected in sterile tubes, rapidly frozen in liquid nitrogen, and then transferred to a −80 °C freezer for subsequent analysis.

### RNA extraction, library construction and sequencing

Total RNA was extracted via a TRIzol reagent kit (Invitrogen, Carlsbad, CA, USA) according to the manufacturer’s protocol. RNA quality was assessed via an Agilent 2100 Bioanalyzer (Agilent Technologies, Palo Alto, CA, USA) and confirmed via RNase-free agarose gel electrophoresis. Following RNA extraction, eukaryotic mRNA was enriched via oligo (dT) beads. The enriched mRNA was then fragmented into short fragments via fragmentation buffer and reverse transcribed into cDNA via the NEBNext Ultra RNA Library Prep Kit for Illumina (NEB #7530, New England Biolabs, Ipswich, MA, USA). The purified double-stranded cDNA fragments were end-repaired, a base was added, and the fragments were ligated to Illumina sequencing adapters. The ligation reaction mixture was purified via AMPure XP beads (1.0X). Ligated fragments underwent size selection through agarose gel electrophoresis and were then amplified via polymerase chain reaction (PCR). Three biological replicates were randomly selected from each group of rats for the following sequencing experiment. The resulting cDNA library was sequenced via an Illumina NovaSeq 6000 system by Gene Denovo Biotechnology Co. (Guangzhou, China).

### Transcriptomic Data processing

Raw reads obtained from sequencing machines contained adapters or low-quality bases, which could impact subsequent assembly and analysis. Therefore, to obtain high-quality clean reads, the data were filtered via fastp (version 0.18.0)^18^. The filtering parameters were as follows: 1) removal of reads containing adapters; 2) removal of reads with more than 10% unknown nucleotides (N); and 3) removal of low-quality reads containing more than 50% low-quality bases (Q value ≤ 20). Short reads were aligned to the ribosomal RNA (rRNA) database via Bowtie2 (version 2.2.8)^19^. Reads mapped to rRNA were subsequently removed, and the remaining clean reads were used for assembly and gene abundance calculation. An index of the reference genome was created, and paired-end clean reads were mapped to the reference genome via HISAT2 (version 2.4)^20^ with “-rna-strandness RF” and other parameters set to defaults. The mapped reads of each sample were assembled via StringTie v1.3.1^21,22^ via a reference-based approach. For each transcription region, a Fragments Per Kilobase of transcript per Million mapped reads (FPKM) value was calculated to quantify expression abundance and variations via RSEM software^23^. Correlation of two parallel experiments provides the evaluation of the reliability of experimental results as well as operational stability. The correlation coefficient between two replicas was calculated to evaluate repeatability between samples. The closer the correlation coefficient gets to 1, the better the repeatability between two parallel experiments. Principal Component Analysis (PCA) was performed with R package gmodels (http://www.r-project.org/).

### Statistical analysis

Body weight, fasting blood glucose levels and mechanical withdrawal thresholds assessed via the von Frey filament test were compared between the two groups. The normality of the data was first assessed using the Shapiro-Wilk test. The results indicated that all three parameters were normally distributed in both the control and diabetic groups (all *P* > 0.05). Therefore, independent-samples t-tests were used for intergroup comparisons, and data are presented as the mean ± SEM. All statistical analyses were performed using SPSS version 22.0 (SPSS, Inc., Chicago, IL, USA). A two-tailed *P* value < 0.05 was considered statistically significant.

## Data Records

Raw sequencing data have been deposited into the NCBI Sequence Read Archive (SRA) database under BioProject accession number PRJNA1192570^24^, with the detailed correspondence of sequencing libraries, individual read sets and their accession numbers provided in Table 1. The transcriptomic dataset, including raw gene read counts and FPKM values, has been deposited in the NCBI Gene Expression Omnibus (GEO) and is publicly available under accession number GSE322631^25^. The transcriptomic dataset has also been deposited in figshare^26^.

**Table 1:** Sequencing libraries, GEO/SRA/BioSample accessions for RNA-seq data of the central amygdala in rats.

Experimental Group	GEO Sample Accession	Library ID	SRA Run Accession	BioSample Accession	Sample Label
Control (rep1)	GSM9555834	c_4	SRR31560019	SAMN45115539	c4
Control (rep1)	GSM9555833	c_5	SRR31560018	SAMN45115540	c5
Control (rep1)	GSM9555835	c_6	SRR31560017	SAMN45115541	c6
Diabetic (rep1)	GSM9555838	s_3	SRR31560016	SAMN45115542	s3
Diabetic (rep2)	GSM9555837	s_4	SRR31560015	SAMN45115543	s4
Diabetic (rep3)	GSM9555836	s_5	SRR31560014	SAMN45115544	s5

## Technical Validation

### Characteristics of the animal model

**Fig. 1:**
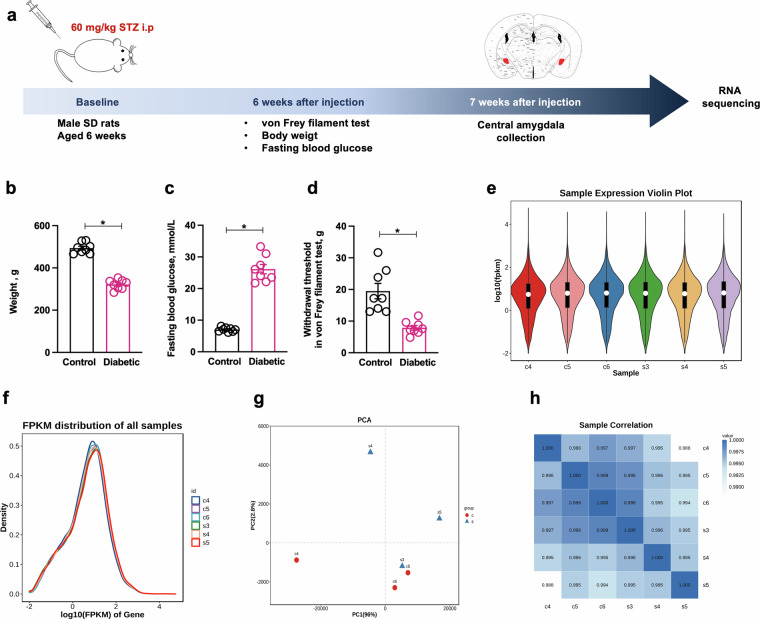
Study design and expression characteristics of the transcriptome. (**a**) Study design: A single dose of 60 mg/kg STZ was intraperitoneally injected into male SD rats. Rats with blood glucose levels greater than 16.7 mmol/l were selected for further study. Six weeks after STZ injection, calibrated von Frey filaments were used, and the rats were weighed and fasted for blood glucose testing. At the end of the experiment, the central amygdala was collected, and total RNA was extracted. The cDNA library was sequenced via an Illumina NovaSeq 6000 platform. (**b**–**d**) Body weight, fasting blood glucose, and mechanical withdrawal threshold in control and diabetic rats. Data are presented as mean ± SEM. Statistical analysis was performed using independent-samples t-tests. * indicates *P* < 0.05. (**e,f**) Gene expression abundance. (**g**) Principal component analysis. (**h**) Correlation heatmap analysis. c: control group; s: diabetic group.

STZ injection successfully induced diabetes in rats, as compared with the control group (Fig. 1a, Table 2). This was evidenced by a significant reduction in body weight [diabetic vs. control: 288.52 ± 8.23 g vs. 441.61 ± 9.00 g, *P* < 0.001; Fig. 1b] and a marked increase in fasting blood glucose levels [diabetic vs. control: 23.70 ± 1.49 mmol/L vs. 6.31 ± 0.23 mmol/L, *P* < 0.001; Fig. 1c]. Six weeks after STZ injection, diabetic rats exhibited behavioral signs of neuropathic pain, as indicated by a significant decrease in the mechanical withdrawal threshold in the von Frey test [diabetic vs. control: 7.88 ± 0.75 g vs. 19.48 ± 2.44 g, *P* < 0.001; Fig. 1d].

**Table 2:** *In vivo* experimental data of rats.

Experimental Group	Replicate	Withdrawal threshold on the von Frey test	Weight (g)	Fasting blood glucose (mmol/L)
Control	c1	13	476.4	7.6
Control	c2	26	530.3	7
Control	c3	23.8	465.7	8.1
Control	c4	17.2	465.6	7.3
Control	c5	14	502.2	6.1
Control	c6	17.2	484.8	6.5
Control	c7	13	528	6.4
Control	c8	31.7	496	7.1
Diabetic	s1	7	305.4	26.1
Diabetic	s2	11.7	337.4	33.3
Diabetic	s3	8.8	302.3	24
Diabetic	s4	6.4	340	31
Diabetic	s5	4.8	331.1	23.5
Diabetic	s6	7.2	283.8	27.4
Diabetic	s7	9.6	353.9	21.7
Diabetic	s8	7.6	319.5	22.1

### Quality control of transcriptome sequencing and gene analysis

To ensure the quality of sequencing data, we performed filtering of low-quality reads to obtain clean reads. As shown in Table 3, the average proportion of clean data was 99.61% (range: 99.57–99.67%), with an average adapter content of 0.03% (range: 0.03–0.04%) and an average low-quality read proportion of 0.36% (range: 0.30–0.39%). Subsequently, we analyzed the base quality of the clean reads. The average proportion of AF_Q20 (bases with quality score ≥ 20) was 97.22% (range: 97.12–97.30%), and the average proportion of AF_Q30 (bases with quality score ≥ 30) was 92.41% (range: 92.20–92.61%). The proportion of AF_N (ambiguous bases) was 0 across all samples, and the average AF_GC (guanine-cytosine content) was 48.25% (range: 47.54–48.66%). These results indicate that the sequencing data exhibited high base quality. We further conducted sequence alignment analysis. For rRNA mapping, the average proportion of mapped reads was 1.71% (range: 1.25–2.22%), suggesting that rRNA contamination had been effectively reduced to an appropriate level. We used the HISAT2 software for alignment against the reference genome. The average proportion of total mapped reads was 95.37% (range: 93.14–96.24%), indicating high sequencing data quality and good consistency with the reference genome. In the alignment statistics for reference regions, the average proportion of reads mapped to exon regions was 76.28% (range: 75.72–76.68%), to intron regions was 6.55% (range: 6.25–7.08%), and to intergenic regions was 17.18% (range: 16.88–17.39%).

**Table 3:** Quality control of transcriptomic data.

Sample	Clean Reads	Q20 (%)	Q30 (%)	GC (%)	rRNA (Bowtie2) Mapped (%)	Reference Genome (HISAT2) Mapped (%)	Reference Genome (HISAT2) Uniq Mapped (%)
c4	38368218	97.23	92.45	47.54	2.11	96.23	89.92
c5	39189850	97.23	92.44	48.66	1.25	96.19	90.42
c6	35952854	97.3	92.61	48.33	1.61	96.24	90.35
s3	48511898	97.12	92.2	48.29	1.56	94.29	88.51
s4	46277156	97.17	92.29	48.13	2.22	93.14	87.3
s5	45728352	97.29	92.46	48.54	1.52	96.1	90.2

A total of 17,116 known genes were detected in the sequencing, accounting for 77.62% of the reference genome. The average proportion of genes with 80–100% coverage was 78.61% (range: 77.48–79.35%, Table 4).

**Table 4:** Detection of known genes and distribution of gene coverage percentages.

Sample	Sequenced Total Genes Number (%)	0–20% Gene coverage (%)	20–40% Gene coverage (%)	40–60% Gene coverage (%)	60–80% Gene coverage (%)	80–100% Gene coverage (%)
c4	15578 (70.65%)	4.70	5.03	5.36	7.43	77.48
c5	15619 (70.83%)	4.44	5.05	4.96	7.14	78.41
c6	15602 (70.76%)	4.45	4.95	5.22	7.10	78.28
s3	15614 (70.81%)	4.42	4.87	4.73	6.77	79.21
s4	15720 (71.29%)	4.16	4.86	4.98	7.11	78.90
s5	15654 (70.99%)	4.34	4.43	4.83	7.06	79.35

### Expression characteristics of the transcriptome

Read alignment to the reference genome was performed using HISAT2, followed by transcript reconstruction with StringTie and gene expression quantification with RSEM. Gene expression levels were calculated as FPKM values for each sample. Expression distribution plots were constructed to characterize the global transcriptomic profiles across all samples (Fig. 1e,f). PCA was performed to assess the global distribution and similarity of all samples based on genome-wide gene expression profiles. The PCA scatter plot (Fig. 1g) presents the distribution of individual biological replicates; each dot represents one sample, and the x- and y-axes correspond to principal component 1 (PC1) and principal component 2 (PC2), which represent the top two sources of variance in the transcriptomic data. Samples from the control and diabetic groups are labeled with distinct colors for visualization. Pearson correlation coefficients were calculated between samples to assess the consistency and repeatability of gene expression profiles across all biological replicates (Fig. 1h).

## Usage Notes

This dataset provides a transcriptomic resource of the CeA in a rat model of diabetes-associated neuropathic pain. This transcriptomic resource can support investigations on the central mechanisms of PDN.

Several limitations of this dataset should be considered for future reuse. First, the RNA-seq analysis includes only three biological replicates per group, which may limit the statistical power of subsequent differential expression analysis. Second, only male rats were included, so the dataset does not capture potential sex differences in CeA transcriptomic profiles related to diabetic neuropathic pain. Third, CeA samples were collected at a single time point (six weeks post-STZ injection), providing a static snapshot of transcriptomic alterations rather than dynamic changes during disease progression.

All raw and processed data in this study are publicly available in standard public repositories, as detailed in the Data Records section, and can be accessed and reused in compliance with the corresponding repository data usage policies.

## Data Availability

No custom code was used to generate or process the data described in this manuscript.
